# Monotherapy Versus Combination Therapy in Agitation Management in the Intensive Care Unit: A Narrative Review

**DOI:** 10.7759/cureus.98201

**Published:** 2025-11-30

**Authors:** Riseethan Kirishnaventhan, German Corso, Suneet Patel, Sarah Batakanwa

**Affiliations:** 1 Medicine, Saint James School of Medicine, The Quarter, AIA; 2 Child and Adolescent Psychiatry and Pediatric Psychiatry, Tropical Texas Behavioral Health, Harlingen, USA

**Keywords:** agitation management, antipsychotics, benzodiazepines, combination therapy, medical intensive care unit

## Abstract

Agitation, defined as excessive restlessness or psychomotor activity leading to potential harm or disruption, is a common and challenging complication in the intensive care unit (ICU), often associated with delirium, withdrawal syndromes, or environmental stressors. Monotherapy refers to the use of a single pharmacologic agent (commonly benzodiazepines or antipsychotics), whereas combination therapy involves the concurrent use of both classes. Agitation contributes to adverse outcomes, including prolonged mechanical ventilation, unintentional removal of intravenous lines, feeding tubes, or endotracheal tubes*. *This can increase morbidity in rare cases, such as patients with difficult airways who can self-extubate, which may increase mortality. This narrative review evaluates evidence from 2000 to 2025, comparing monotherapy and combination therapy in ICU agitation. Although benzodiazepines remain essential for alcohol or sedative withdrawal, their broader use is associated with higher delirium incidence and longer ICU stay. Antipsychotics are widely used but have not consistently demonstrated improvement in delirium outcomes in randomized trials. Combination therapy may provide rapid behavioral control in refractory or mixed etiology agitation, but it appears to increase the risk of oversedation, hypotension, and prolonged mechanical ventilation. Current critical care guidelines, including the 2018 Pain, Agitation/Sedation, Delirium, Immobility, and Sleep Disruption (PADIS) recommendations, do not recommend routine combination therapy; instead, they emphasize individualized patient selection, structured sedation protocols, and cautious titration. Clinical decision-making should balance immediate control of agitation with long-term neurologic and functional outcomes.

## Introduction and background

Agitation is a frequently observed clinical manifestation in critically ill patients in intensive care units (ICUs). It is defined as a state of excessive psychomotor activity, irritability, restlessness, and, in severe cases, aggression or combativeness, often resulting in interruption of care [1]. Monotherapy refers to the use of a single medication class, such as a benzodiazepine or an antipsychotic, whereas combination therapy involves the concurrent use of both drug classes. Agitation in the ICU can interfere with essential medical interventions such as mechanical ventilation (which typically requires concurrent sedation), intravenous line placement, and hemodynamic monitoring - all of which are critical to the clinical stability of these patients [2]. Moreover, agitation increases healthcare resource utilization and is associated with adverse outcomes, including prolonged ICU stay, accidental extubation, and rare cases of increased mortality and morbidity(e.g., self-extubation in patients with a difficult airway where timely re-intubation is not possible). Because of the high prevalence of agitation in the ICU, optimal pharmacologic treatment remains an ongoing debate among healthcare providers, particularly regarding the choice between monotherapy and combination therapy.

Agitation is one of the most common and difficult symptoms to manage in critically ill patients, with the ICU being a frequent setting. It is most often induced by delirium, drug withdrawal, underlying psychiatric illness, pain, or environmental factors [1,2]. Effective management of agitation is crucial, as it may lead to complications such as self-extubation, the need for increased sedation, prolonged ICU stay, and even higher mortality.

Pharmacologic therapy with benzodiazepines, antipsychotics, or a combination of both is commonly used to manage agitated patients [3]. However, the optimal regimen remains uncertain. Some institutions prefer monotherapy protocols, while others initiate combination therapy early in treatment. Both drug classes have distinct mechanisms and adverse effects, and their simultaneous use raises concerns about oversedation, prolonged delirium, and increased ICU length of stay [4].

This narrative review aims to explore recent literature comparing monotherapy and combination therapy (using benzodiazepines and antipsychotics) for managing agitation in the ICU. The review examines utilization patterns, proposed benefits and risks, and clinical outcomes associated with each strategy. Its purpose is to determine whether combination therapy provides additional clinical benefit over monotherapy or simply adds to polypharmacy and its risks. A narrative review approach was chosen over a systematic review because the literature on this topic varies widely in methodology and scope. This format allows the integration of diverse study types and expert perspectives.

Research methods

This article is a narrative review summarizing recent evidence and expert opinions regarding the use of monotherapy and combination therapy for agitation management in the ICU, incorporating findings from diverse study designs and clinical contexts.

Types of Studies

Randomized controlled trials, observational cohort studies, case-control studies, systematic reviews and meta-analyses, case reports or case series, narrative reviews, expert opinions, and clinical guidelines.

Participants

Adult patients (≥18 years) admitted to medical, surgical, or mixed ICUs who received pharmacologic treatment for agitation with benzodiazepines, antipsychotics, or both.

Search Strategy

Databases searched included PubMed, Embase, and the Cochrane Library. Search terms were: ICU OR intensive care, agitation OR agitated, delirium, benzodiazepines OR lorazepam OR midazolam, antipsychotics OR haloperidol OR quetiapine, monotherapy, and combination therapy. Relevant MeSH terms and Boolean operators were used to optimize the search.

Intervention Type

Pharmacologic management of agitation using benzodiazepines, antipsychotics, or both.

Outcome Measures

Duration and severity of agitation, incidence of oversedation or delirium, ICU length of stay, mortality, and the need for restraints or reintubation.

Language

Only studies published in English were included.

Timeframe

Studies published between January 2000 and July 2025 were reviewed.

Study Selection

Article selection occurred in two phases: (1) title and abstract screening for relevance to ICU agitation management, and (2) full-text review to confirm inclusion based on predefined criteria. Reference lists of included articles and recent reviews were also screened to identify additional eligible studies. Inclusion and exclusion criteria were documented.

Quality Assessment

The methodological quality of key studies was assessed using validated tools such as the Cochrane Risk of Bias tool for randomized controlled trials and the Newcastle-Ottawa Scale for observational studies, particularly when results were used to support specific recommendations.

Grading the Evidence

No formal grading system was applied. Instead, this review comments on the strengths and weaknesses of available evidence, data quality, and consistency of findings when comparing monotherapy and combination therapy.

Epidemiology and clinical impact of ICU agitation

Between 20% and 60% of ICU patients experience agitation, with prevalence exceeding 80% among those with underlying delirium or substance withdrawal [5]. The risk of agitation is higher in surgical ICU patients, elderly individuals, and those with cognitive impairment [6,7]. Agitation contributes to multiple complications, including increased infection risk from restraint use, higher sedation requirements, and disruption of normal sleep-wake cycles [8]. It is also strongly associated with delirium, which predicts long-term cognitive decline and higher six-month mortality [9]. Agitated patients often require prolonged mechanical ventilation and have a greater risk of developing ventilator-associated pneumonia [10]. Prompt recognition and management of agitation are essential to improving patient outcomes.

Etiology and pathophysiology of agitation

The causes of agitation in the ICU are varied and often interrelated, including physiological, neurological, pharmacological, and environmental factors [11]. Delirium is the most frequent cause, accounting for up to 80% of agitation in mechanically ventilated ICU patients, even though these patients are already sedated; in such cases, delirium is understood as a contributing, not a primary, cause of agitation [12]. Delirium may arise from infection, metabolic derangements, organ failure, pain, sedative exposure, or withdrawal from alcohol or benzodiazepines [13]. These processes disrupt neurotransmitter balance within the central nervous system. Pathophysiologically, agitation is thought to involve dysregulation of the cholinergic and dopaminergic systems: acetylcholine deficiency impairs attention and memory, while increased dopaminergic activity contributes to psychosis, hallucinations, and restlessness [14]. Glutamate-mediated excitotoxicity via overactive N-methyl-D-aspartic acid (NMDA) receptors can exacerbate neuronal injury and arousal in severe cases [15]. Additionally, pro-inflammatory cytokines such as interleukin-6 and tumor necrosis factor-alpha can cross the blood-brain barrier, altering synaptic activity and promoting delirium and cognitive dysfunction [16]. Neuroimaging studies have demonstrated cortical atrophy and metabolic changes in the frontal lobe and hippocampus among patients who develop ICU delirium. Sleep deprivation - common in the ICU due to constant lighting, noise, and frequent staff interactions - disrupts circadian rhythms and melatonin secretion, further increasing agitation risk [17]. These overlapping mechanisms make agitation common and challenging to manage in critically ill patients.

## Review

Assessment and diagnosis

Proper assessment of agitation in the ICU is central to ensure timely and appropriate management of agitation. The Richmond Agitation-Sedation Scale (RAAS) is the most widely used tool by providers for assessing agitation and sedation levels in critically ill patients [17]. The RAAS scale provides a numeric score ranging from -5 (unarousable) to +4 (combative), which allows for the standardization of monitoring of these patients. The Confusion Assessment Method for the ICU (CAM-ICU) is commonly used to assess for delirium, a frequent underlying cause of agitation [18]. Another tool that can be used is the Intensive Care Delirium Screening Checklist (ICDSC) and the Sedation-Agitation Scale (SAS), which can also help standardize the severity of patient agitation [19]. Clinical evaluation should include a thorough medical history of medications, alcohol and drug use, metabolic disorders, and psychiatric illness. Diagnostic testing generally involves laboratory tests, imaging, and EEG in cases of suspected seizures or encephalopathy [20].

Pharmacologic treatment strategies

Treatment of ICU agitation depends on an integration of non-pharmacologic and pharmacologic treatment plans. Non-pharmacologic interventions such as reorientation, adjusting lighting, early mobilization, and proper sleep hygiene are crucial first-line treatments for patients with agitation [21]. But if these first-line therapies do not work, behavior is the one that is usually managed first with the help of pharmacologic therapies [22]. Benzodiazepines and antipsychotics are used most, although other agents like dexmedetomidine, ketamine, and valproate are increasingly used in certain circumstances [23]. Adjusting sedation to the cause of agitation and considering patient comorbidities, risk of oversedation, and neurologic status is required. Sedation guidelines and daily interruption of sedation have been shown to reduce ICU length of stay and should become standard of care [24].

Antipsychotics in agitation management

Typical antipsychotics (e.g., haloperidol) and atypical antipsychotics (e.g., quetiapine, olanzapine) are used for the management of delirium and agitation in the ICU. The main mechanism is blockade of the Dopamine D2 receptor, but the atypical also block serotonin and histamine receptors as well [25]. Despite widespread usage, there is not a great deal of strong evidence to indicate their effectiveness in agitation management. In the MIND-USA (Modifying the Impact of Delirium in the ICU - USA) trial, it has been shown that haloperidol and ziprasidone were no more effective than placebo in reducing the duration of delirium [26]. However, antipsychotics remain part of standard practice due to their known safety and control of agitation. Adverse effects may still take place, such as QT interval prolongation, extrapyramidal side effects, and sedation, and patients need to be monitored with ECG and dose titration [27]. Atypical antipsychotics may be better tolerated, but head-to-head comparisons in ICU patients are limited, and current evidence regarding their effect on delirium outcomes remains inconclusive.

Benzodiazepines in agitation management

The mechanism of action of benzodiazepines is to increase the duration of their effects on gamma-aminobutyric acid (GABA-A) receptors in the central nervous system [25]. This will result in sedation, anticonvulsant activity, anxiolysis, and muscle relaxation due to the inhibitor effects of GABA. In the ICU setting, lorazepam and midazolam are the two most commonly used medications because of the preferred route of intravenous administration [26]. Benzodiazepines is the treatment of choice for agitation caused by alcohol or benzodiazepine withdrawal because of reduced GABAergic tone [27]. In these cases, the benzodiazepine can be considered lifesaving and can decrease seizures and death in these patients. Outside withdrawal syndromes, the application of benzodiazepines is associated with noteworthy side effects. In recent observational studies and randomized trials (e.g., MENDS (Maximizing the Efficacy of Sedation and Delirium Management Study), SEDCOM (Safety and Efficacy of Dexmedetomidine Compared With Midazolam)), it has been shown that patients receiving lorazepam experience increased rates of ICU delirium, prolonged mechanical ventilation, and longer ICU stays when compared with other agents like dexmedetomidine [28,29]. Midazolam, due to its lipid solubility and hepatic metabolism, is particularly susceptible to accumulation in the context of hepatic dysfunction [30]. Lorazepam, although hydrophilic, has been shown to carry the risk of propylene glycol toxicity in prolonged infusions [31]. Benzodiazepines also cause respiratory depression, especially in patients who are receiving a combination of opioids and benzodiazepines. Benzodiazepines can also worsen respiratory depression in patients who have impaired respiratory reserve despite such risks. But they are still frequently used because they are effective, cheap, and familiar to healthcare providers. Its use in agitation must be carefully monitored for withdrawal symptoms or for specific cases of short-term sedation only [30,31].

Rationale behind combination therapy

In many ICU settings, benzodiazepines and antipsychotics are administered in combination to rapidly control the severity of agitation [32]. This practice is best known for its advantages of targeting multiple neurochemical pathways simultaneously. Benzodiazepines act on the GABAergic system, while antipsychotics target dopaminergic and serotonergic pathways [33]. Combination therapy may be particularly useful in patients with overlapping withdrawal and delirium or in cases of acute agitation unresponsive to monotherapy. However, the sedative effect raises concerns for respiratory depression, prolonged mechanical ventilation, and oversedating the patient [34]. Due to this potential risk, guidelines typically do not advise its use in routine combination therapy, which is most often based on the clinical judgement and experience of the provider rather than strong empirical evidence [35,36].

Clinical evidence: monotherapy versus combination therapy

Although clinical experience and practice trends suggest a rationale for using combination pharmacologic agents for agitation, evidence supporting the superiority of combination therapy over monotherapy remains limited, as shown in Table 1. The concurrent use of benzodiazepines and antipsychotics theoretically allows clinicians to target both the GABAergic and dopaminergic systems, offering potentially synergistic control of agitation in the ICU. However, only a small number of studies have directly investigated this strategy.

**Table 1:** Summary of the representative studies comparing single-agent strategies versus concurrent use of benzodiazepines and antipsychotics for agitation or delirium-associated agitation in ICU populations. Abbreviations: BZD: benzodiazepine; LOS: length of stay; RCT: randomized controlled trial.

Author & Year	Design	Sample Size	Intervention	Key Findings	Limitations	Favored Approach
Tomichek JE et al., 2016 [31]	Prospective cohort	710	Antipsychotic exposure during ICU stay and at discharge	Antipsychotics frequently ↑in ICU; no clear benefit on delirium outcomes	No standard protocol	Monotherapy
Girard TD et al., 2018 (MIND-USA) [29]	RCT	566	Haloperidol / ziprasidone vs placebo	No reduction in delirium duration	Limited power for subgroups	Monotherapy
Pandharipande PP et al., 2007 (MENDS) [4]	RCT	106	Dexmedetomidine vs lorazepam	↓ delirium days; ↓ mortality trend	Excluded severe psychosis	Non-BZD monotherapy
Riker RR et al., 2009 (SEDCOM) [13]	RCT	375	Dexmedetomidine vs midazolam	↓ delirium; shorter LOS	Not agitation-specific	Monotherapy
Devlin JW et al., 2010 [30]	Pilot RCT	36	Quetiapine + haloperidol vs haloperidol	Faster delirium resolution	Underpowered	Combination (cautious)
Hughes CG et al., 2012 [23]	Cohort	225	Combination vs single agent	↑ ventilation time with combination	Non-randomized	Monotherapy
Fraser GL et al., 2013 [3]	Systematic review / meta-analysis	15 RCTs	BZD vs non-BZD sedation	BZD → ↑ delirium	Heterogeneous dosing	Non-BZD monotherapy

In a retrospective cohort study by Tomichek et al., the use of haloperidol plus lorazepam was not associated with a reduction in ICU length of stay or a decreased duration of agitation compared with monotherapy using either agent [31]. Furthermore, patients receiving combination therapy exhibited higher rates of oversedation and more frequent episodes of hypotension, suggesting an unfavorable risk-benefit ratio. Similarly, a secondary analysis of the Brain-ICU Study found that combination sedative regimens were independently associated with worse long-term neurocognitive outcomes, raising concern that deeper or prolonged sedation may contribute to persistent cognitive impairment after ICU discharge [37].

Randomized controlled data provide further perspective. The MENDS trial compared dexmedetomidine and lorazepam monotherapy in mechanically ventilated ICU patients and demonstrated that dexmedetomidine was associated with fewer days of delirium, lower mortality trends, and shorter duration of ventilation [28]. The SEDCOM study yielded similar findings, reporting that patients sedated with dexmedetomidine experienced significantly less delirium and shorter ICU stays than those receiving midazolam [29]. Although neither trial directly evaluated combination therapy, their outcomes collectively emphasize that limiting benzodiazepine exposure - and maintaining lighter, goal-directed sedation - leads to improved neurologic recovery.

The Modifying the Impact of Delirium in the ICU - USA (MIND-USA) trial, one of the largest placebo-controlled studies in this area, compared haloperidol and ziprasidone with placebo for delirium treatment in ICU patients [26]. It found no difference in the duration of delirium or survival outcomes between groups, suggesting that dopamine blockade alone is unlikely to reverse delirium-related agitation. This finding challenges the assumption that combining antipsychotics with benzodiazepines would meaningfully improve outcomes.

A small pilot study by Devlin et al. evaluated quetiapine plus haloperidol versus haloperidol monotherapy in agitated ICU patients and observed faster symptom resolution in the combination arm [30]. However, this study’s limited sample size and lack of standardized sedation targets make its results difficult to generalize. Case reports and small case series similarly suggest possible short-term benefits of combination therapy for severe agitation, particularly in mixed-etiology cases involving withdrawal and delirium, but such evidence remains low-quality and inconsistent.

**Figure 1:**
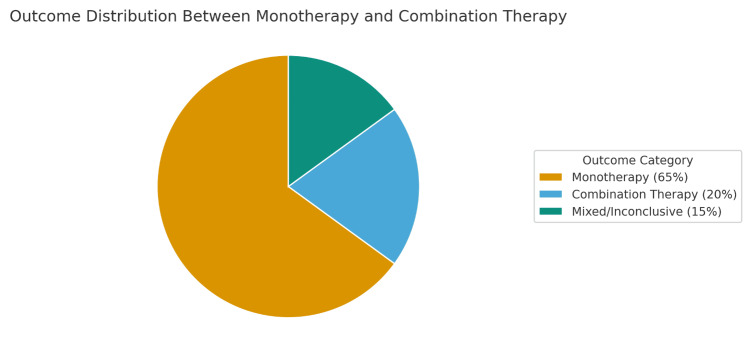
A pie chart illustrating the proportion of studies reporting more favorable clinical outcomes with each strategy. Approximately 65% of studies favored monotherapy, 20% favored combination therapy, and 15% showed no significant difference.

**Figure 2:**
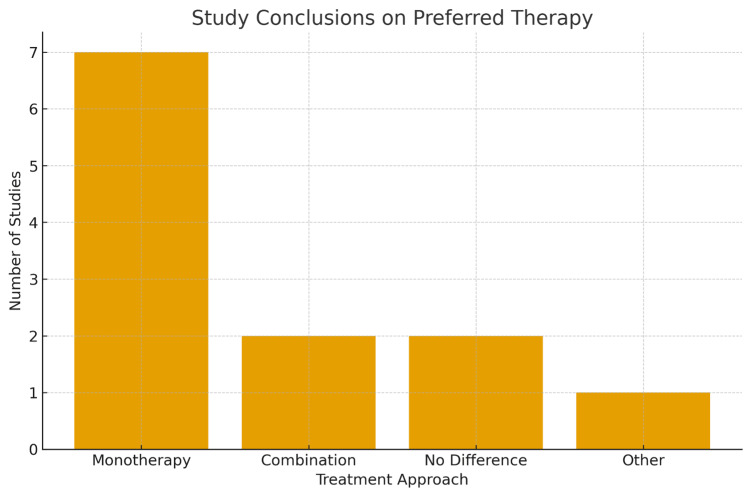
A bar chart depicting the number of studies concluding in favor of monotherapy, combination therapy, no difference, or other alternatives. Most available data support monotherapy.

Taken together, the body of literature indicates that while combination therapy may occasionally be necessary in refractory cases, it does not appear to shorten delirium duration, reduce ICU length of stay, or improve survival when compared to optimized monotherapy, as shown in Figures 1, 2. Moreover, combination therapy may increase the risk of oversedation, hemodynamic instability, and delayed extubation. The 2018 Pain, Agitation/Sedation, Delirium, Immobility, and Sleep Disruption (PADIS) guidelines align with this evidence, recommending benzodiazepine minimization and discouraging the routine use of dual-agent therapy except in specific, clinically justified contexts. Given the emphasis on sedation minimization and neuroprotection in modern critical care, there remains a pressing need for large, multicenter trials to determine whether any subset of ICU patients benefits from combination therapy beyond short-term behavioral control.

Guidelines and clinical practice patterns 

Management of agitation in the ICU is guided by several professional society recommendations, most notably the 2018 PADIS Guidelines issued by the Society of Critical Care Medicine (SCCM) [38]. These guidelines emphasize minimizing benzodiazepine exposure in favor of non-benzodiazepine sedatives such as dexmedetomidine or propofol, which have been shown to reduce delirium duration and mechanical ventilation time. The PADIS panel explicitly discourages the routine use of antipsychotics for the prevention or treatment of delirium, except in cases where agitation places the patient or staff at risk of harm [39]. Similarly, routine use of combination therapy - benzodiazepine plus antipsychotic - is not recommended due to the absence of robust evidence supporting improved outcomes and the potential for increased adverse effects.

Despite these recommendations, real-world clinical practice remains variable. Surveys of ICU clinicians continue to show frequent reliance on benzodiazepines and haloperidol, especially in centers where access to alternative agents such as dexmedetomidine may be limited or cost-prohibitive [40]. Institutional culture, prescriber familiarity, and nursing comfort levels often influence drug selection as much as evidence-based protocols. Some ICUs have adopted structured sedation protocols emphasizing light sedation targets and daily interruption, while others still practice deeper continuous sedation due to staffing or resource constraints.

The discrepancy between guideline recommendations and bedside practice reflects both logistical and educational challenges. Providers may face uncertainty when managing mixed-etiology agitation - such as cases involving both delirium and withdrawal - where guideline-directed monotherapy may not adequately control symptoms. In such situations, clinicians often revert to combination therapy out of necessity rather than preference. This underscores the importance of ongoing education, protocol standardization, and multidisciplinary collaboration (between intensivists, pharmacists, and nursing teams) to promote guideline adherence while maintaining individualized patient care. Ultimately, current guidelines converge on several key principles such as the following: (i) sedation minimization: Use the lightest effective dose and titrate to objective scales (e.g., RASS, SAS); benzodiazepine avoidance outside withdrawal contexts, antipsychotic use only for acute behavioral control, not delirium prevention, and nonpharmacologic interventions (e.g., sleep hygiene, reorientation, mobilization) should be first-line in all cases. Until stronger data clarify the role of dual therapy, adherence to PADIS-based protocols and local quality improvement initiatives remain the most evidence-aligned approach to ICU agitation management.

Safety, risks, and monitoring

The pharmacologic management of ICU agitation carries significant risks that necessitate close and continuous monitoring. Benzodiazepines are associated with respiratory depression, hypotension, and an increased incidence of delirium [41]. Their sedative properties can also delay ventilator weaning and prolong ICU length of stay. These effects are compounded in patients receiving concomitant opioids or other central nervous system depressants. Antipsychotics can cause electrocardiographic (ECG) abnormalities such as QT interval prolongation and torsades de pointes, as well as neurologic adverse events including extrapyramidal symptoms (EPS) and neuroleptic malignant syndrome (NMS) [42]. Patients prescribed these agents, especially at high doses or over prolonged periods, should undergo baseline and follow-up ECGs to monitor for cardiac arrhythmias and QTc changes.

In combination therapy, the simultaneous use of benzodiazepines and antipsychotics further amplifies these risks. Dual therapy has been linked to increased oversedation, prolonged mechanical ventilation, and delayed extubation compared to monotherapy [43]. The additive depressant effects on the respiratory and cardiovascular systems can produce hemodynamic instability and impair spontaneous breathing trials [42]. In addition, deep or prolonged sedation resulting from combination regimens may contribute to long-term neurocognitive impairment and post-ICU syndrome, emphasizing the importance of targeted and reversible sedation strategies.

To mitigate these risks, standardized monitoring tools such as the Richmond Agitation-Sedation Scale (RASS) and the Sedation-Agitation Scale (SAS) should be applied at regular intervals to titrate medications to the lightest effective level of sedation [17,18]. Daily sedation interruptions, when clinically appropriate, help minimize drug accumulation and facilitate neurological assessment. Laboratory monitoring should include renal and hepatic function tests for drug clearance, electrolyte evaluation for QT prolongation risk, and serum drug levels when applicable [27]. Interdisciplinary collaboration between physicians, pharmacists, and nursing staff is essential for early identification of adverse effects and timely medication adjustments.

Ultimately, vigilant monitoring and adherence to sedation protocols can significantly reduce iatrogenic complications associated with pharmacologic management of ICU agitation [20]. Continuous reassessment ensures that sedation depth aligns with the patient’s evolving clinical status, maintaining a balance between safety, comfort, and neurologic recovery.

Gaps in the literature and research limitations

There remains a critical need for more rigorous research directly comparing monotherapy and combination therapy in the management of ICU agitation. The existing literature is limited by small sample sizes, single-center study designs, and inconsistent outcome measures. Many investigations have been retrospective or observational, which limits causal inference and increases the risk of selection bias. Furthermore, study populations vary widely with respect to agitation etiology, sedation protocols, and baseline patient characteristics, making cross-study comparisons difficult and reducing external validity.

Few studies adequately account for confounding factors such as concomitant opioid or sedative use, underlying delirium subtypes, and comorbid psychiatric or withdrawal syndromes, all of which influence both drug selection and outcomes. Additionally, most published studies have short follow-up periods, focusing on acute ICU outcomes such as agitation duration or mechanical ventilation time, while neglecting long-term measures like cognitive function, post-discharge quality of life, and cost-effectiveness. These omissions limit understanding of how pharmacologic strategies influence recovery beyond the ICU.

Randomized controlled trials specifically evaluating combination therapy are scarce, and many exclude complex patients such as those with polypharmacy, alcohol withdrawal, or multi-organ dysfunction - precisely the populations most likely to receive combination treatment in clinical practice. This exclusion further reduces generalizability and may underestimate the potential adverse effects of these regimens. Moreover, variability in sedation depth targets, dosing strategies, and assessment tools (e.g., RASS, CAM-ICU) contributes to heterogeneity and inconsistent findings across studies.

Lastly, there is limited reporting on adverse event surveillance and standardized safety endpoints, such as QT prolongation, hypotension, or time to extubation, which are essential for evaluating risk-benefit trade-offs. Future studies must incorporate standardized definitions and uniform sedation protocols to ensure meaningful comparisons. Until then, conclusions regarding the superiority or safety of monotherapy versus combination therapy remain tentative.

Future research directions

Future research should aim to clarify the optimal pharmacologic approach for managing agitation in critically ill patients. Large, multicenter randomized controlled trials are needed to compare monotherapy and combination therapy using standardized definitions of agitation, consistent sedation targets, and objective outcome measures. Studies should also include diverse ICU populations, such as patients with alcohol withdrawal, psychiatric comorbidities, or multi-organ dysfunction, to improve generalizability. Incorporating long-term follow-up to assess post-ICU cognitive recovery, quality of life, and healthcare utilization will strengthen understanding of downstream effects. In addition, emerging tools such as neuroimaging, biomarker analysis, and pharmacogenomic profiling may help identify which patients benefit most from specific sedative strategies. Ultimately, a coordinated research effort emphasizing methodological consistency and patient-centered outcomes is essential to guide evidence-based practice in ICU agitation management.

Discussion

In clinical practice, management of ICU agitation should prioritize non-pharmacologic strategies, including reorientation, sleep promotion, and early mobilization, while reserving pharmacologic therapy for situations in which agitation poses a safety threat to the patient or healthcare staff [21]. Clinicians must individualize treatment choices based on comorbidities, delirium risk, and institutional resources [22]. ICU agitation management has been studied across diverse patient populations, with evidence describing outcomes and safety considerations for benzodiazepines, antipsychotics, and combination therapy [25,26,35,36]. Until stronger data emerge, minimizing sedation while maintaining safety remains the most effective strategy for optimizing outcomes in ICU agitation management.

The findings from this review have important implications for clinical practice in the ICU setting. Patient risk profiles and underlying etiology must be considered when developing a treatment plan for agitation, given the widespread use of benzodiazepines and antipsychotics. Benzodiazepines remain the treatment of choice for alcohol or benzodiazepine withdrawal, but outside these indications, their association with delirium, oversedation, and prolonged ventilation warrants caution. Antipsychotics are commonly used for agitation related to delirium; however, randomized trials such as MIND-USA have failed to demonstrate significant benefits, underscoring the need for individualized prescribing [25,26,35,36]. Combination therapy may still be reasonable in select cases, particularly among patients with overlapping withdrawal and delirium or those with refractory agitation unresponsive to single agents. Nonetheless, the increased risk of oversedation, hemodynamic instability, and long-term cognitive impairment necessitates a careful balance between therapeutic efficacy and patient safety. Continuous monitoring using standardized sedation scales (e.g., RASS, SAS) and adherence to structured sedation protocols are essential for minimizing adverse outcomes.

## Conclusions

In conclusion, agitation in the ICU is a prevalent and potentially harmful condition that requires timely recognition and effective management. While both benzodiazepines and antipsychotics are widely used, the optimal strategy, whether combination or monotherapy, remains uncertain. Current evidence does not support the routine use of combination therapy over monotherapy, as it has not been shown to improve clinical outcomes and may increase adverse effects such as oversedation and prolonged ventilation. Clinical decisions should therefore be guided by each patient’s underlying etiology, comorbidities, and risk profile, with a careful balance between therapeutic benefit and potential harm. Greater adherence to evidence-based sedation protocols, including regular reassessment and use of standardized tools such as RASS or SAS, is essential to minimize complications and promote neurologic recovery.

Moving forward, continued research and collaborative clinical efforts are necessary to refine treatment algorithms and identify patient subgroups who may benefit from targeted therapies. Until stronger evidence emerges, adopting a conservative, protocol-driven approach that prioritizes patient safety, minimizes unnecessary sedation, and integrates non-pharmacologic interventions remains the most effective strategy for improving outcomes and standardizing care across ICUs.
